# Comparative Genome Analysis of Genes Regulating Compound Inflorescences in Tomato

**DOI:** 10.3390/ijms222212548

**Published:** 2021-11-21

**Authors:** Yahui Yang, Huanhuan Yang, Yinxiao Tan, Tingting Zhao, Xiangyang Xu, Jingfu Li, Jingbin Jiang

**Affiliations:** Laboratory of Genetic Breeding in Tomato, Key Laboratory of Biology and Genetic Improvement of Horticultural Crops (Northeast Region), Ministry of Agriculture and Rural Affairs, College of Horticulture and Landscape Architecture, Northeast Agricultural University, Harbin 150030, China; yyh201296@126.com (Y.Y.); huanyaya0126@sina.com (H.Y.); 186153xx@sina.com (Y.T.); 15504506671@sohu.com (T.Z.); xxy709@126.com (X.X.)

**Keywords:** tomato, compound inflorescence, RNA-seq, differentially expressed genes

## Abstract

Inflorescences are the main factor affecting fruit yield. The quantity and quality of inflorescences are closely related to fruit quality and yield. The presence of compound inflorescences in cherry tomatoes is well established, and it has been discovered by chance that compound racemes also exist in tomatoes. To explore the formation of compound inflorescences in tomato, transcriptome sequencing was performed on Moneymaker (MM) and Compound Inflorescence (CI) plants. In-florescences were collected in three periods (early, middle and late) in three replicates, for a total of 18 samples. Data analysis showed that the DEGs were most enriched in metabolic pathways and plant hormone signal transduction pathways. The DEGs were also enriched in the cell cycle pathway, photosynthesis pathway, carbon metabolism pathway and circadian rhythm pathway. We found that the *FALSIFLORA* (*FA*), *COMPOUND INFLORESCENCE* (*S*) and *ANANTHA* (*AN*) genes were involved in compound inflorescence development, not only revealing novel genes but also providing a rich theoretical basis for compound inflorescence development.

## 1. Introduction

Tomato (*Solanum lycopersicum* L.) is one of the most important agricultural cash crops in the world [1]. It is a typical plant with a syntactic axial mode of growth [2,3,4] and has become a model plant for the study of plant reproduction and development. The inflorescence structure of tomato varies greatly, ranging from a single branching inflorescence to dozens of branching inflorescences. Inflorescence branch number (BN) is an important characteristic that determines the final fruit number of each inflorescence and affects crop yield [5].

The inflorescences of tomato follow a pattern of synaxial development, and their growth and development are accompanied by the interplay between vegetative growth and reproductive growth [6]. Complex racemes have been found in cherry tomatoes and in other plants, such as *Pisum sativum*, *Medicago truncatula* and *Lotus japonicus* [7,8,9]. In contrast, *Arabidopsis thaliana* and *Antirrhinum majus* show simple racemes [10,11]. The two determinants of inflorescence structure are internode form and pedicel characteristics [12,13]. Likewise, many important genes involved in the regulation of inflorescence development have been identified, such as the *SELF-PRUNING* (*SP*) [13], *FALSIFLORA* (*FA*) [14], *COMPOUND INFLORESCENCE* (*S*) [15], *ANANTHA* (*AN*) [16], *UNIFLORA* (*UF*) [17], *TERMINATING FLOWER* (*TMF*) [18], *SINGLE FLOWER TRUSS* (*SFT*) [19], *MACROCALYX* (*MC*) [20], *JOINTLESS* (*J*) [21], *BLIND* (*BL*) [22], and *LATERAL SUPPRESSOR* (*LS*) genes [23]. Many transcription factors, such as *LFY*, *AP1*, *TFL1*, *SOC1*, *FA*, *SFT*, *AN*, *TMF* and *S*, play important roles in the developmental regulation of the inflorescence meristem and flower meristem in both *Arabidopsis thaliana* and tomato [24,25,26]. For example, either *AN* or *FA* causes highly branched inflorescence structures. However, *AN* and *FA* differ in causing either the loss of flower-forming ability or unrestricted lateral syntaxis meristem formation, leading to the cauliflower-type (*AN*) and vegetative-type (*FA*) development [27]. *S* mutants can still produce flowers, but the rate of transformation from the inflorescence meristem to the flower meristem is slow, and only one flower is formed for every 2–4 inflorescence meristems, which ultimately leads to the formation of complex inflorescences [28]. *S* encodes Wuschel-related HOMEOBOX 9 (*WOX9*) [29], and *AN* and *FA* encode the F-box ubiquitous ORGANS (UFO) protein and a transcription factor, respectively. LEAFYS is mainly expressed in the primary inflorescence meristem to control its transformation to the flower meristem [30]. However, *AN* and *FA* are mainly expressed in the flower meristem and control the formation of the flower meristem [19,31]. In contrast to *S*, *AN* and *FA*, early flowering (*TMF*) mutants show a raceme inflorescence structure [30]. In *TMF* mutants, flower meristem determinants such as *AN* and *FA* are prematurely expressed in the stem apical meristem, causing loss of the ability to form a new synaxeme meristem. *TMF* encodes a member of the *Arabidopsis LIGHT-SENSITIVE HYPOCOTYL 1*, Oryza G1 (ALOG) protein family expressed at the apex of the stem whose primary function is to maintain vegetative growth and prevent premature flowering [30]. *Joinless* (*J*) is a *MADS-box* gene mutant that causes plants to shift to vegetative growth after 1–3 flowers are formed in an inflorescence. J is mainly expressed in the inflorescence meristem to prevent it from returning to vegetative growth and the premature transformation of the flower meristem [19].

Inflorescences are the main factor that determines fruit yield, and the quantity and quality of inflorescences are directly related to the quality and yield of fruit. The presence of complex racemes in cherry tomatoes is well established and was also found by chance in ordinary tomatoes. In this experiment, we compared RNA-seq results and DEG roles to explore whether the complex racemes of common tomato are regulated by similar genes to the complex racemes of cherry tomato. The clarification of the rules of inflorescence development provides an important theoretical basis for formulating breeding objectives and cultivation management methods and achieving high, optimal yields by controlling the growth and development of inflorescences and fruits.

## 2. Results

### 2.1. RNA Sequencing Data and Functional Analysis

**Figure 1:**
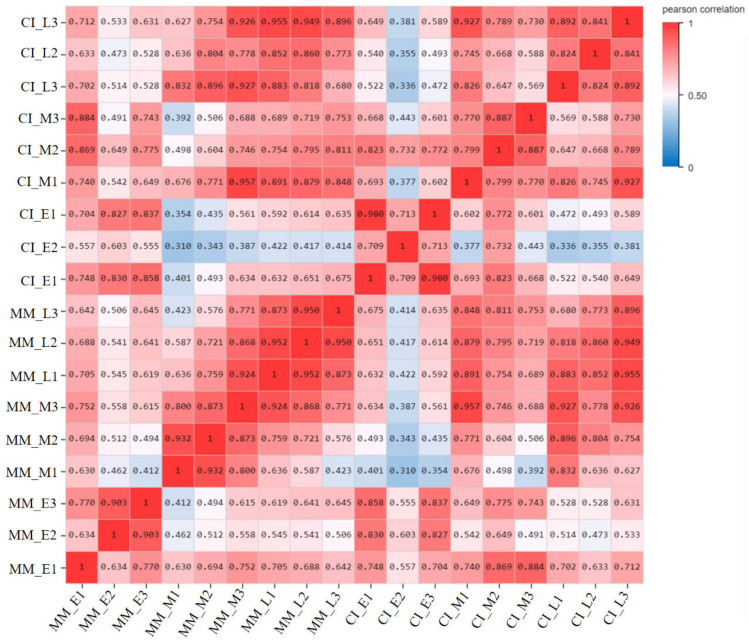
Pearson correlation coefficients of the 18 samples. The correlation coefficients between two samples are visualized as heat maps.

To obtain transcriptome data for the two tomato materials with different inflorescence, we completed transcriptome sequencing during the inflorescence from early, middle and late stages. The average ratios of the genomes and gene sets were 93.53% and 83.97%, respectively. A total of 23,634 genes were detected. The Illumina mass fraction 20 (Q 20) and 30 (Q 30) values represent the percentages of sequencing data with error rates of less than 1 or 0.1%, respectively (Cock et al., 2010). In this study, more than 97% of reads met the Q 20 criterion, and 92% of clean reads reached the Q 30 threshold (Table 1). Only the data with a Q 30 quality were used for subsequent analysis. At least 92% of the reads were mapped to the tomato reference genome, and more than 90% of the clean reads were among the mapped reads. Pearson correlation coefficients were calculated for all gene expression values between each pair of samples, and the correlation coefficients of 18 data sets (23,634 genes in total) are presented as heat maps (Figure 1). These charts reflect the correlation of gene expression among the samples, with the more similar the levels of gene expression are, the higher the correlation coefficients will be.

### 2.2. Expression and Analysis of Differentially Expressed Genes (DEGs) in Tomato Inflorescences

**Figure 2:**
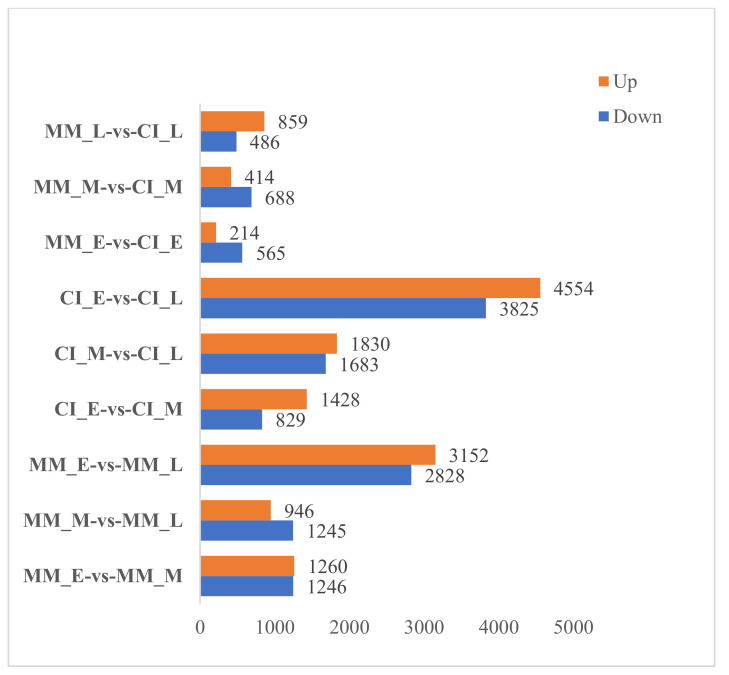
Statistics of the DEGs among different comparison groups.

**Figure 3:**
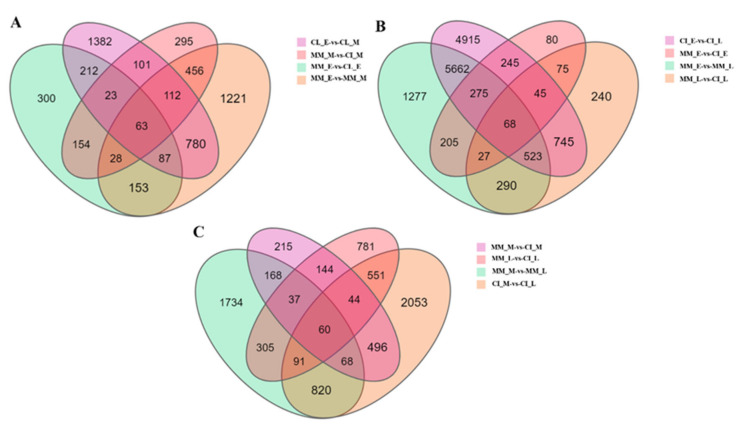
Venn diagram of 9 groups of DEGs. (**A**) Venn diagram of DEGs identified in the MM_E vs. MM_M, CI_E vs. CI_M, MM_E vs. CI_E and MM_M vs. CI_M comparisons. (**B**) Venn diagram of DEGs identified in the MM_E vs. CI_E, MM_L-vs. CI_L, MM_E vs. MM_L and CI_E vs. CI_L comparisons. (**C**) Venn diagram of DEGs identified in the MM_M vs. MM_L, CI_M vs. CI_L, MM_M vs. CI_M and MM_L vs. CI_L comparisons. Each circle represents a set of genes, and the overlapping regions of different circles represent the coexpressed genes.

According to Figure 2, CI_E vs. CI_L showed the most obvious upregulation and downregulation of DEGs, including 4554 upregulated genes and 3525 downregulated genes, followed by MM_E vs. MM_L in which 3152 DEGs were significantly upregulated and 2828 were significantly downregulated. In MM_E vs. CI_E, 214 DEGs were upregulated and 565 were downregulated, and in MM_M vs. CI_M, 414 DEGs were upregulated, and 688 DEGs were downregulated. The results showed that the changes in the expression of DEGs in the inflorescences during different stages were more obvious than those in the two plants during each period. However, we still believe that it is more meaningful to study the changes in DEGs in the two plants during the same period. The Venn diagram of DEGs shows the number of DEGs identified in each comparison group and the overlap between the comparison groups. A total of 63 genes were identified in the early and middle stages of MM and CI plants, 68 genes were identified in the early and late stages of MM and CI plants, and 60 genes were identified in the middle and late stages of MM and CI plants. These common genes may be closely related to the development of complex inflorescences (Figure 3).

### 2.3. Gene Ontology (GO) Functional Classification and Analysis of DEGs

**Figure 4:**
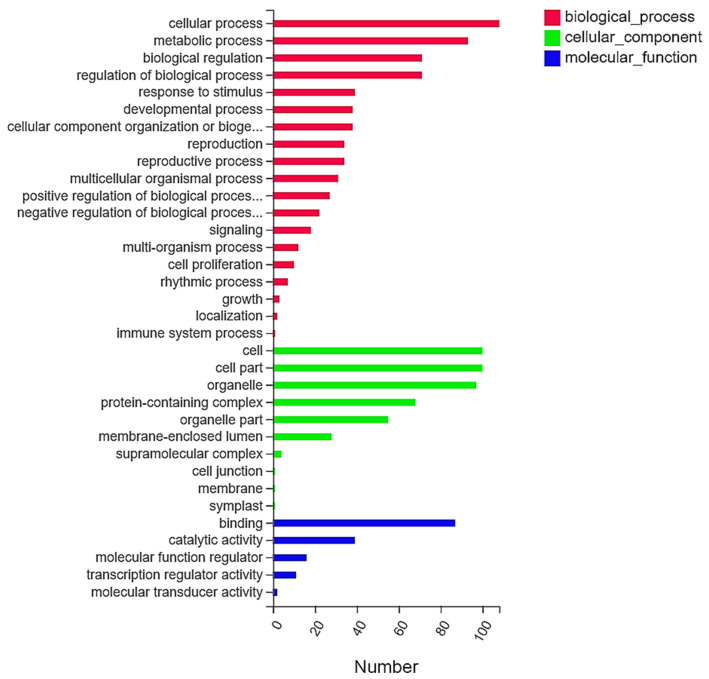
Gene ontology categories of DEGs.

The GO database is a comprehensive database describing gene functions. The GO enrichment analysis results were divided into three main functional categories: molecular function, cellular process and biological process (Q 0.05) (Figure 4). The greatest number of DEGs were enriched in biological processes and participated in cellular processes, metabolic processes, biological regulation, biological process regulation, development and stimulus responses (Q 0.05). The cell cycle, metabolic processes and biological regulation were also significantly enriched. The enriched cell processes included cells, organelles, cell parts, protein complexes and organelle parts. (Q 0.05). Notably, among the DEGs, many genes were significantly enriched in biological process categories, including the cell cycle (108 DEGs), metabolism (93 DEGs), and biological regulation (71 DEGs) (Table S2).

### 2.4. Kyoto Encyclopedia of Genes and Genomes (KEGG) Enrichment Analysis of DEGs

**Figure 5:**
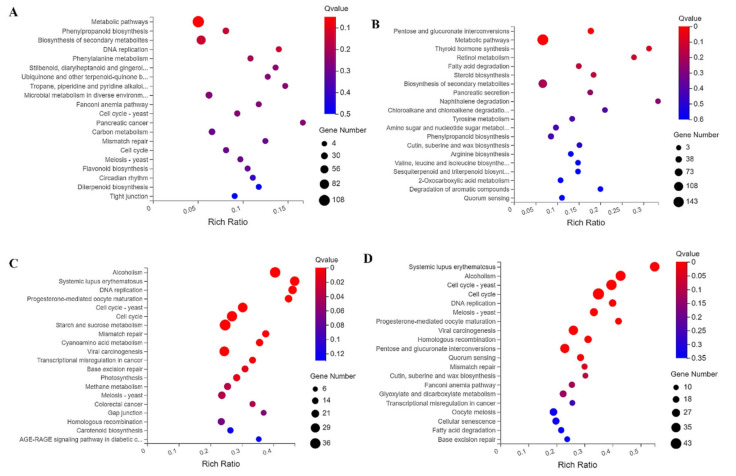
Bubble diagram of KEGG pathway enrichment. (**A**) Enrichment of DEG KEGG pathways in the MM_E vs. CI_E comparison. (**B**) Enrichment of DEG KEGG pathways in the MM_M vs. CI_M comparison. (**C**) Enrichment of DEG KEGG pathways in the MM_E vs. MM_M comparison. (**D**) Enrichment of KEGG pathways of DEGs in the CI_E vs. CI_M comparison. The sizes of the bubbles indicate the numbers of genes enriched in the KEGG pathways, where the larger the bubble is, the greater the number of genes, and the color of the bubble represents the Q value.

By analyzing KEGG pathways, we can understand the detailed biological functions of genes. As shown in Figure 5, in the MM_E vs. CI_E group, DEGs involved in metabolic pathways (108 DEGs), plant hormone signal transduction (11 DEGs), the cell cycle (10 DEGs), amino sugar and nucleotide sugar metabolism (9 DEGs) and circadian rhythms (5 DEGs) were significantly enriched (Figure 5A and Table S2A). In the MM_M vs. CI_M comparison, DEGs involved in metabolic pathways (143 DEGs), plant hormone signal transduction (15 DEGs), amino sugar and nucleotide sugar metabolism (10 DEGs), the cell cycle (7 DEGs), and circadian rhythms (2 DEGs) showed the greatest enrichment (Figure 5B and Table S2B). In the MM_E vs. MM_M group, the DEGs were significantly enriched in the cell cycle (43 DEGs), starch and sucrose metabolism (23 DEGs), cellular senescence (18 DEGs) and carotenoid biosynthesis (8 DEGs) pathways (Figure 5C and Table S2C). In the CI_E vs. CI_M group, the cell cycle (33 DEGs), starch and sucrose metabolism (36 DEGs) and carotenoid biosynthesis (10 DEGs) pathways were significantly enriched (Figure 5D and Table S2D). The results showed that compared with materials with different inflorescence traits in the same period (early or middle), DEGs were significantly enriched in the metabolic, plant hormone signal transduction, cell cycle and circadian rhythm pathways. Compared with the same material at different stages (early or middle), DEGs were significantly enriched in the cell cycle, starch and sucrose metabolism and carotenoid biosynthesis pathways. Therefore, from the four comparison groups, we can conclude that metabolic pathways, plant hormone signal transduction, the cell cycle and circadian rhythms are involved in regulating the development of complex inflorescence traits in tomato. In addition, the cell cycle, starch and sucrose metabolism and carotenoid biosynthesis are involved in the growth and development of tomato inflorescences.

### 2.5. Screening and Analysis of Genes Affecting Inflorescence Traits in Tomato

In transcriptome analysis, determining whether a transcript is differentially expressed in different samples is one of the core components of the analysis. Forty-one candidate genes related to complex tomato inflorescence traits were selected from the MM_E vs. CI_E and MM_M vs. CI_M groups by combining the gene expression, log2-fold change, KEGG and GO data (Table 2). Fourteen upregulated DEGs were associated with metabolic pathways. The *FZY4* gene plays an important role in growth and development through its contribution to the local free auxin pool. Both *ACO2* and *CAB-3C* participate in the TCA cycle; *ACO2* is related to mitochondria, and *CAB-3C* is related to chloroplasts. The *LIN6*, *CHI3* and *TPX2* enzymes are involved in physiological and biochemical reactions in plants. Both *DFR* and *AnthOMT* are involved in anthocyanin synthesis. *PSY2* is expressed throughout tomato development. *PSY2* is a second tomato gene encoding phytoene synthase. *PLDa1* and *SlCMT3* are involved in many processes during tomato development. *PMK2* enhances biological resistance, *GSH1* participates in vernalization, and *GABA-TP3* participates in reproductive development. Thirteen DEGs were associated with plant hormone signal transduction, all of which were upregulated. *SlCYP735A2-TP3* is associated with an increase in transcription levels after flowering, and its gene expression is high from preanthesis to early postanthesis and is associated with cytokinins. Two gibberellin-related genes, *GA20ox1* and *GA2ox3*, were identified. Seven auxin-related genes were identified, including *IAA17*, *IAA35*, *GH3-4*, *SLAX5*, and *SIGH3.4*. Three ethylene-related genes, *EREB*, *FUL2*, *ERF68*, were found. Exogenous salicylic acid treatment of *LOC100191111*-susceptible plants increases *PR1* gene activity and leads to the development of resistance to nematode invasion in these plants. Three of the DEGs were involved in the cell cycle pathway, and their expression was upregulated. Other candidate genes were not classified into specific pathways but also played important roles in regulating inflorescence development and the formation of different inflorescence traits in tomato plants. *Ls* was detected in tomato inflorescences and can inhibit the growth of tomato side branches. *TMF*, whose expression was upregulated, can coordinate the flowering process by synchronizing flower formation with the gradual reproductive transition, which in turn plays a key role in determining the formation of simple and complex inflorescences. Two identified *MADS-box* genes are inflorescence development regulators that are related to inflorescence characteristics and are involved in changes in floral development mechanisms related to floral structure fixation. In the *BLIND* mutant of tomato, the initiation of the lateral meristem is blocked during stem and inflorescence development, resulting in a significant decrease in inflorescence number. Therefore, the upregulated expression of the *BLIND* gene may promote compound inflorescence formation. *TM29* changes flower morphology, causing tomato petals and stamens to be green instead of yellow and stamens and ovaries to be sterile, after which unisexual fruits develop, so downregulated *TM29* expression leads to normal flower structure development. Therefore, *TM29* plays a role in flower organ development, fruit development and the maintenance of flower meristem characteristics in tomato. *S* encodes transcription factors, and *AN* encodes f-box proteins that control inflorescence structure by promoting the continuous stages of inflorescence meristem development to control flower size. *S*, *AN* and *FA* tomato mutants show highly branched inflorescence structures. However, the loss of flower-forming ability associated with *AN* and *FA* results in the unrestricted formation of the lateral axonal meristem, leading to cauliflower-type (*AN*) for vegetative-type (*FA*) development. However, the transformation rate from the inflorescence meristem to the flower meristem is slower in the *S* mutant, and only one flower forms from every 2 to 4 axonal meristems, resulting in the formation of complex inflorescences. In the MM_E vs. CI_E and MM_M vs. CI_M groups, the expression of *S*, *FA* and *AN* was upregulated, indicating that *S*, *FA* and *AN* were produced in large quantities in early and middle stages, while the expression of MM_L-vs. CI_L was downregulated, indicating that *S*, *FA* and *AN* were mutated in late stages, leading to the formation of complex inflorescences (Table 2 and Table S3). In conclusion, some genes associated with metabolic pathways, plant hormone pathways and cell cycle pathways as well as genes not related to pathways were significantly changed, which may be important reasons for the formation of complex inflorescences in tomato.

### 2.6. MapMan Tool-Based Analysis of DEGs

**Figure 6:**
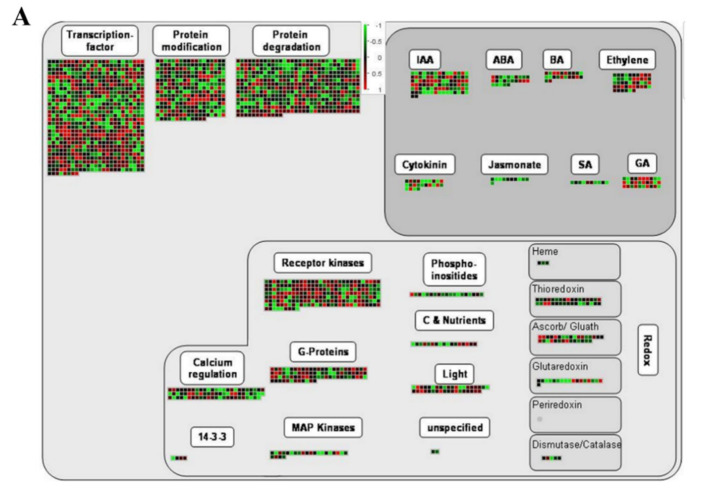

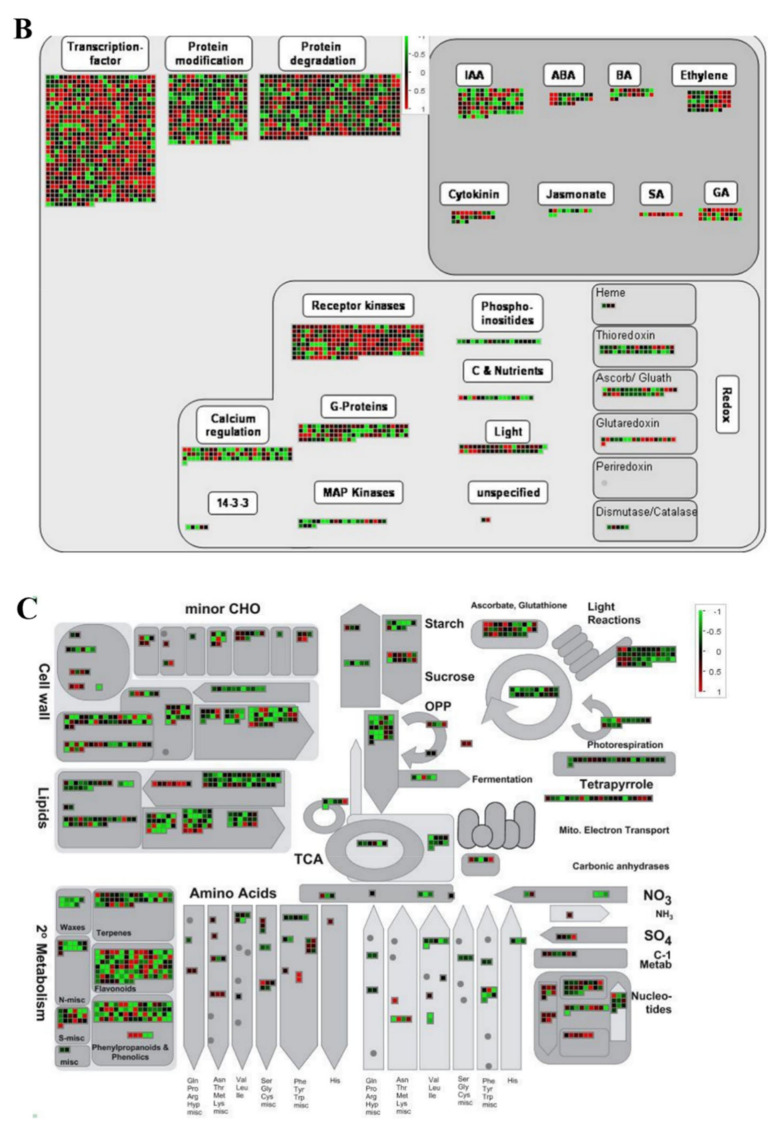

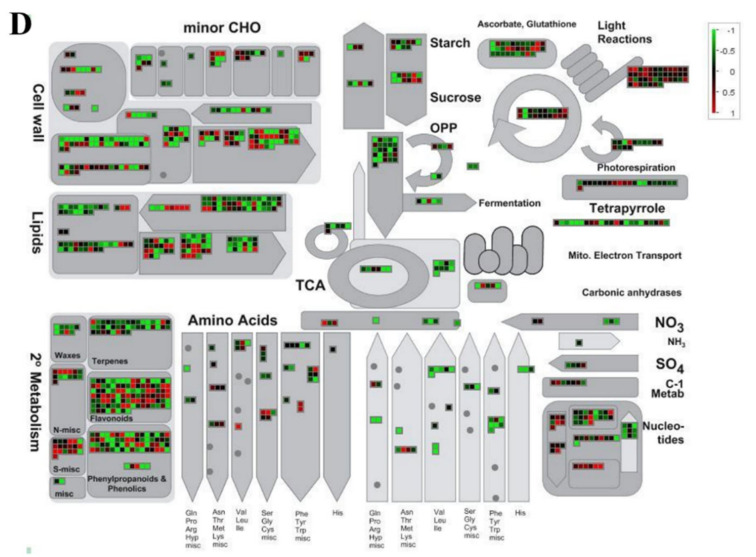
Clustering pattern of DEGs generated using the MapMan tool. (**A**) Biological regulation of DEGs in MM_E vs. CI_E. (**B**) Biological regulation of DEGs in MM_M vs. CI_M. (**C**) Metabolic regulation of DEGs in MM_E vs. CI_E. (**D**) Metabolic regulation of DEGs in MM_M vs. CI_M.

To better understand how different inflorescence traits are generated in tomato, effective data were extracted from the MM_E vs. CI_E and MM_M vs. CI_M groups. (Figure 6). From the biological regulatory pathway analysis (Figure 6A,B), we can conclude that more transcription factors were upregulated among MM_M vs. CI_M DEGs than the MM_E vs. CI_E DEGs, but the two groups showed similar expression related to protein modification and protein degradation. The results showed that the DEGs mainly regulated the hormones indoleacetic acid (IAA), abscisic acid (ABA), brassinosteroids (BR), ethylene, cytokinin (CTK), jasmonic acid (JA), salicylic acid (SA) and gibberellin (GA). The DEG expression results of MM_E vs. CI_E and MM_M vs. CI_M were similar, but MM_M vs. CI_M showed more upregulated genes than MM_E vs. CI_E. As shown in Figure 6A,B, the expression of genes involved in the IAA pathway was highest, while the expression of genes involved in the SA pathway was lowest. In addition, there were significantly more upregulated genes than downregulated genes in the GA pathway. The DEGs of the M_E vs. CI_E and MM_M vs. CI_M groups were also enriched in REDOX reactions. The DEGs were most enriched in receptor kinases but also in biological reactions regulated by calcium, G proteins, MAP kinase, photorespiration, carbon, nutrients, and light. According to the analysis of metabolic pathways (Figure 6C,D), DEGs related to the cell wall, lipids, secondary metabolism, amino acids, TCA, starch, sucrose, light reactions, enrichment of photorespiration and nucleotides were upregulated, and more genes showed decreased than increased expression. Most of the genes were enriched in secondary metabolism, and more genes were downregulated than upregulated. The MM_M vs. CI group exhibited more DEGs than the MM_E vs. CI_E group, which were related to the respiration process. It can be concluded from the above results that bioregulatory and metabolic pathways play an important role in the regulation of tomato inflorescence traits and that plant hormones also play an important role in tomato inflorescence traits.

### 2.7. Weighted Gene Co-Expression Network Analysis (WGCNA) of DEGs

**Figure 7:**
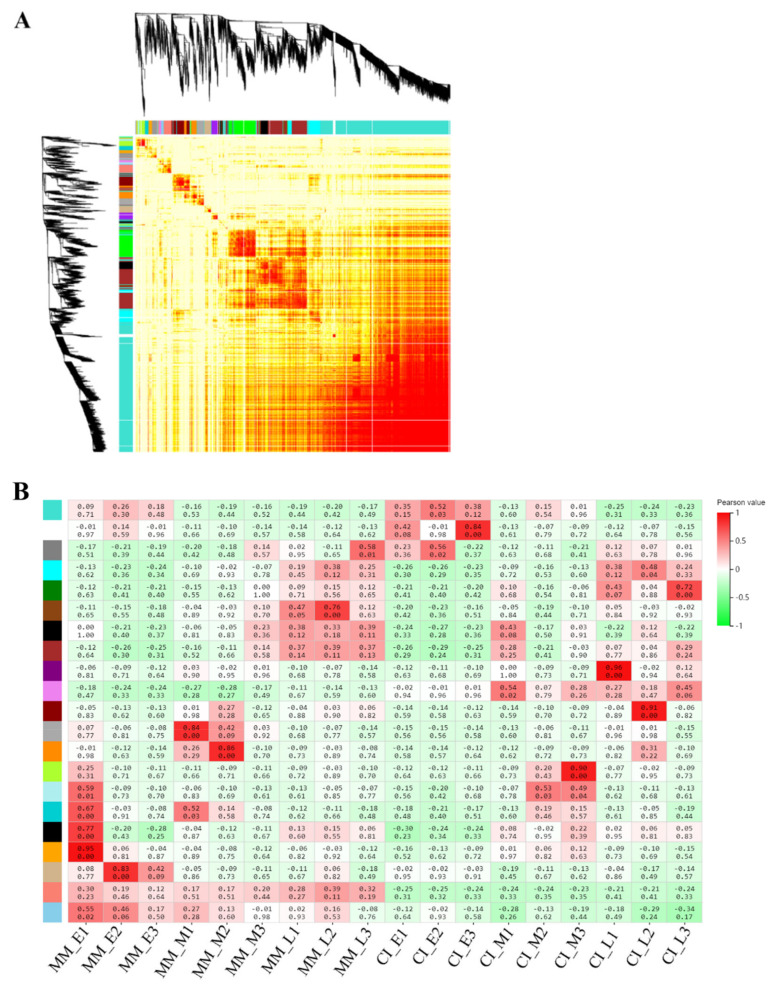
Weighted gene co-expression network analysis (WGCNA). (**A**) Different colors represent different gene modules, indicating different coefficients between genes. (**B**) Coexpression and correlations between the gene modules and phenotypes. The abscissa represents the samples; the ordinate represents the modules. The upper value in each cell represents the correlation coefficient, and the lower value represents the *p*-value.

**Figure 8:**
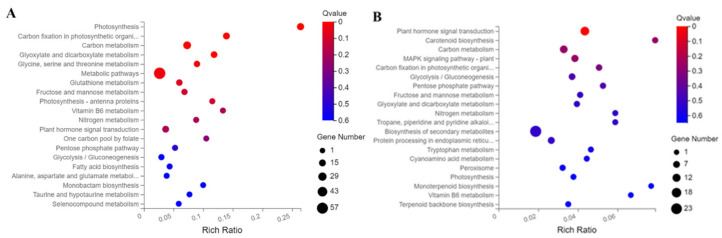
Bubble diagram of enrichment in KEGG pathways. (**A**) Enrichment KEGG pathways of DEGs in the brown module (480 genes). (**B**) Enrichment of KEGG pathways of DEGs in the green module (338 genes).

In WGCNA, gene expression data are used to construct coexpression gene modules. According to the results (Figure 7A), we obtained 21 gene co-expression modules, where a different color represents each module. The turquoise (1786 genes), white (38 genes), grey (1 gene), cyan (215 genes), green (338 genes), saddle brown (35 genes), black (110 genes), brown (480 genes), purple (89 genes), violet (30 genes), dark red (171 genes), dark grey (100 genes), dark orange (111 genes), green-yellow (88 genes), pale turquoise (31 genes), dark turquoise (51 genes), grey (66 genes), orange (40 genes), tan (87 genes), salmon (121 genes), sky blue (37 genes), brown (480 genes) and green (338 genes) genes were highly specific, and their KEGG pathways were analyzed (Figure 7B). The brown (480 genes) KEGG pathway module showed significant enrichment in photosynthesis, carbon fixation, carbon metabolism, and metabolic pathways in photosynthetic organisms (Figure 8A). The green (338 genes) KEGG pathway module showed significant enrichment in plant signaling pathways, carbon metabolism, and carbon fixation during photosynthesis (Figure 8B).

### 2.8. Verification of DEG Expression Patterns

**Figure 9:**
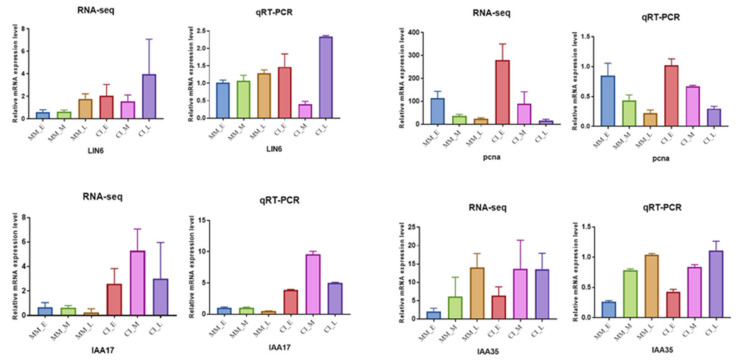

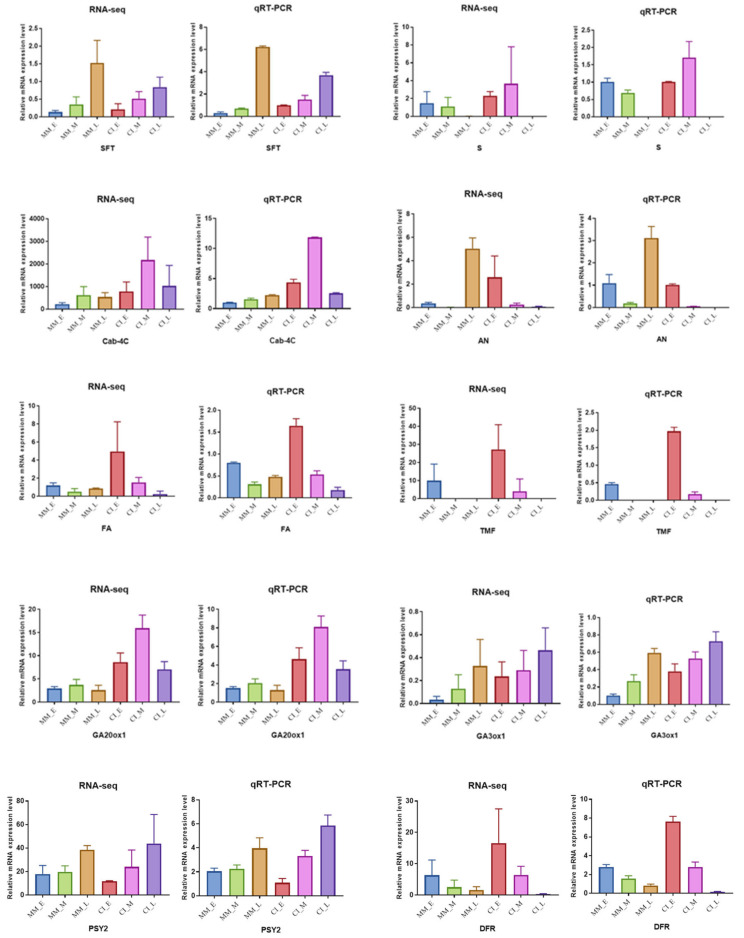
Comparative analysis of expression results between RNA-seq and qRT-PCR for 14 DEGs. Three technical replicates were performed for each biological replicate.

To verify the accuracy of the DEG expression patterns shown by RNA-seq data, we performed qRT-PCR analysis on 14 DEGs with 3 biological replicates (Figure 9). These 14 DEGs were randomly selected from 40 candidate genes that may be involved in the regulation of tomato inflorescence traits. It contains 4 genes of metabolic pathway, 4 genes of plant hormone signaling, 1 gene of cell cycle and 5 genes closely related to compounding inflorescence. The results showed that the gene expression trends of these 14 genes were the same in RNA-seq and qRT-PCR. (Table S4). These results indicate that RNA-seq data are of high quality and can be used for subsequent analysis.

## 3. Discussion

A notable manifestation of plant evolution is the existence of distinct branch and inflorescence traits [32,33]. There is wide variation in inflorescence complexity in Solanaceae, and flowering marks the end of main shoot growth [34,35,36]. Inflorescences are derived from the growth of dome-shaped clusters of pluripotent cells known as apical meristems. The apical meristems first produce leaves, and after flowering induction, they produce inflorescence meristems, which then transition to flower meristems, producing flowers. We used second-generation molecular sequencing technology, RNA-seq, to investigate gene expression and screen candidate genes for prediction. In this study, we sequenced the transcriptomes of two different types of inflorescences (single (MM) and complex (CI)) in three stages (early inflorescences, middle inflorescences, and late inflorescences) and verified the transcriptome data by qRT-PCR. The results showed that the RNA-seq data were reliable.

The transcriptome data showed that there were a large number of DEGs in the 9 comparison groups. We set up three comparison groups between the two materials and found that DEGs were the most abundant in the middle and late inflorescence comparison groups, indicating that the genes regulating inflorescences were highly expressed during the middle to late inflorescence stages. Therefore, there were more DEGs identified in the comparisons of the same materials in different periods. Although differences in the expression of DEGs between the different materials at the same time were smaller, we found that the DEGs identified in these comparisons included more valuable genes. For example, *MADS-box* proteins are one of the most important transcription factor families. The *MADS-box* transcription factor family is a large transcription factor family that is widely distributed in eukaryotes. In higher plants, *MADS-box* transcription factors are mainly involved in the regulation of flower organ formation, flower development, inflorescence development, fruit development and ripening [37,38]. In this study, we identified two *MADS-box* family genes (*MADS-MC* and *MADS1*), and the expression level of *MADS-MC* was downregulated, while the expression level of *MADS1* was upregulated. It was speculated that the upregulated expression of the *MADS1* gene might promote the generation of complex inflorescences, which was consistent with previous studies, while the result for *MADS-MC* was inconsistent with previous studies.

GO enrichment analysis showed that the cell cycle, metabolic processes and biological regulatory pathways were significantly enriched and are involved in the formation of complex inflorescences in tomato. The KEGG results showed that metabolic pathways, plant hormone signal transduction, the cell cycle and circadian rhythms are involved in regulating the development of complex inflorescence traits in tomato. Sucrose metabolism and carotenoid biosynthesis are also involved in the growth and development of tomato inflorescences. Therefore, both the GO enrichment and KEGG enrichment results indicated that metabolic processes, the cell cycle, plant hormone signal transduction and biological regulatory processes (circadian rhythm) are closely related to the formation of composite traits in tomato. We screened genes from metabolic pathways. The *FZY4* gene is involved in the tryptophan metabolic pathway and plays an important role in growth and development through its contribution to local free auxin pools [39]. *ACO2* is involved in cell isolation and the expression of other abscission-related genes associated with programmed cell death, which induces tomato pedicel formation in vitro, and *ACO2* is expressed in both pedicel and adjacent tissues [40]. *ACO2* is related to mitochondria, while *CAB-3C* is related to chloroplasts [40]. *PSY2* is a second tomato gene encoding phytoene synthase, an intermediate in the carotenoid biosynthesis pathway, and is expressed during the development of tomato [41,42]. *SlCMT3* is related to methylation, which is closely related to flowering and other characteristics of tomato plants [43]. Low temperature can stimulate glutathione biosynthesis and cause oxidative stress, and glutathione disulfide (GSSG) is thought to trigger a low-temperature response [44]. Therefore, *GSH1* is involved in vernalization and is closely related to inflorescence development.

Plant hormone signal transduction pathways play an important role in the development of tomato inflorescences. In this study, we selected 14 DEGs and plant hormone signal transduction-related genes, including genes involved in auxin signaling (*IAA17 IAA10*, *GH3-4*, *SLAX5*, *LOC101055555*, and *SIGH3.4*). The temporal and spatial control of auxin distribution plays a key role in the regulation of plant growth and development, and much has been learned about the mechanisms affecting auxin pools and gradients in vegetative tissues [45]. Auxin is a central hormone that has multiple effects on the development of roots, branches, flowers and fruits. The perception and signal transduction of auxin depends on the synergistic action of many components, among which the auxin protein plays a key role. The *IAA* gene shows different expression patterns in different organs and tissues of tomato, and some tissues and organs also present different responses to auxin and ethylene, suggesting that *Aux*/*IAA*s play a role in connecting these two hormone signaling pathways [45]. In contrast, although auxin regulates many aspects of fruit development, *SlCYP735A2-TP3* is associated with increased transcriptional levels after flowering, and from preanthesis to early postanthesis, this gene is highly expressed and related to cytokinin [46]. Two gibberellin-related genes, *GA20ox1* and *GA2ox3*, play a role in regulating organ growth and promoting reproductive development throughout the growth period of crops. Both long and short days require gibberellin to induce flowering to complete reproductive development. In this study, the expression levels of candidate genes (*GA20ox1* and *GA2ox3*) identified in the gibberellic acid pathway were upregulated at the flowering stage. In addition, gibberellin promotes inflorescence development and regulates floral organ formation. Other plant hormones also play a role in regulating the complex network of crop flowering and have a positive effect on the formation of complex inflorescences in tomato.

Bioregulatory and metabolic pathways play key roles in inflorescence development in tomato plants. We used MapMan to analyze DEGs in both biological regulatory and metabolic pathways. Among biological regulatory pathways, we concluded that the DEGs mainly regulated the hormones IAA and cytokinin CTK. When plants are treated with auxin polar transport inhibitors, leaf order patterns change, and lateral organs and flower meristems fail to form, resulting in the development of an acicular inflorescence [47,48]. The pid monopoterous (mp) mutant also exhibits a spiculate phenotype [47,49]. All of these results suggest that the polar transport of auxin can affect the differentiation of inflorescence rachises and flower meristems. Previous studies have shown that auxin is a key regulatory factor in flower development [50] and that the pin1 mutant usually does not flower, suggesting that auxin plays an important role in the formation of flower primordia [51,52]. The study of the auxin signal transduction pathway provides a theoretical basis for better understanding the hormone regulation of inflorescence and floral organ development. Cytokinins are important flower hormones that regulate the initiation of flower primordia and the development of flower organs and participate in the regulation of stamen and pistil development in flowering plants, delaying the aging of flower organs [53,54,55]. The analysis of metabolic pathways showed that DEGs were enriched in the following categories cell wall, lipids, secondary metabolism, amino acids, TCA, starch, sucrose, light reactions, photorespiration and nucleotides. Therefore, we can conclude that biological control approaches and metabolic pathways of tomatoes are important regulators of inflorescence characters and that plant hormones have an important influence on tomato inflorescence properties. In the brown KEGG pathway module identified by WGCNA, significant enrichment was observed in the categories of photosynthesis, carbon fixation of photosynthetic organisms, carbon metabolism, and metabolic pathways (Figure 8A). In the green KEGG pathway module, it was found that the plant signal transduction pathway, carbon metabolism, carbon fixation in photosynthesis, and photosynthesis pathways showed significant enrichment (Figure 9). According to MapMan analysis and WGCNA, it could be concluded that plant hormone signal transduction and metabolic pathways play an important role in regulating inflorescence development in tomato plants. In addition, photosynthesis and carbon metabolism pathways are closely related to inflorescence development.

The mutation of the *S*, *AN* and *FA* genes is the main cause of the formation of compound inflorescences. In previous studies, the observation of many bifurcating inflorescences and single branches of inflorescences were used to identify the BN genes controlling the inflorescences bearing small tomato fruits. The sequence comparison of *FA* and *S* genes and their promoters from two parents showed missense mutations in their coding regions. The authors inferred that SNPs in the coding sequences may cause changes in the functions of *FA* and *S* genes, which may be an important factor determining the formation of BN [2,56]. Molinero-Rosales were the first to identify a tomato *FA* mutant [6]. Tomato *FA* shows homology to *Arabidopsis FLO* and *LFY*. The *Arabidopsis LFY* gene and its homologs encode a major transcription factor that plays a pleiotropic role in the nutrition-to-reproduction transition as well as meristem flower fate, flowering time, and flower arrangement [57,58,59]. Due to the important role of *LFY* in reproductive transformation, even weakly mutated alleles can completely transform at least a few flowers into buds [60]. *FLO*/*LFY* single mutants of dicotyledonous species show increased branching due to flower-to-bud transformation, suggesting that *FLO*/*LFY* proteins inhibit branching by promoting flower development [61,62]. In addition, *LFY* activity is partially dependent on environmental and internal factors, such as light conditions and plant age, and can be induced by exogenous GA treatment [63,64]. Previous studies have shown that *SPL13* positively regulates the expression of *SFT*, a gene related to tomato inflorescences, by directly binding to the *SFT* promoter region and thus controls inflorescence development [65]. The expression of *SFT*, *AN* and *FA* can promote the flowering of tomato [66], and if these genes are mutated, it will delay flowering and result in the formation of complex inflorescences. *SFT* is a homolog of FT in *Arabidopsis* and is downregulated in the circadian pathway (Figure S1). In this study, the expression levels of *SFT*, *S*, *FA*, *GA20ox1* and *GA2ox3* were upregulated in the early comparison group, indicating that *SFT*, *S* and *FA* can promote the flowering of tomato, *GA20ox1* and *GA2ox3* can promote the reproductive development of tomato inflorescences, and complex inflorescence formation involves the circadian rhythm pathway. In addition, the expression levels of *S*, *AN* and *FA* were downregulated in the late comparison group. Hence, it is speculated that the mutation of *S*, *AN* and *FA* in the late stage of tomato inflorescences leads to decreased expression levels and promotes the formation of complex inflorescences, which is consistent with previous studies.

## 4. Materials and Methods

### 4.1. Test Materials

**Figure 10:**
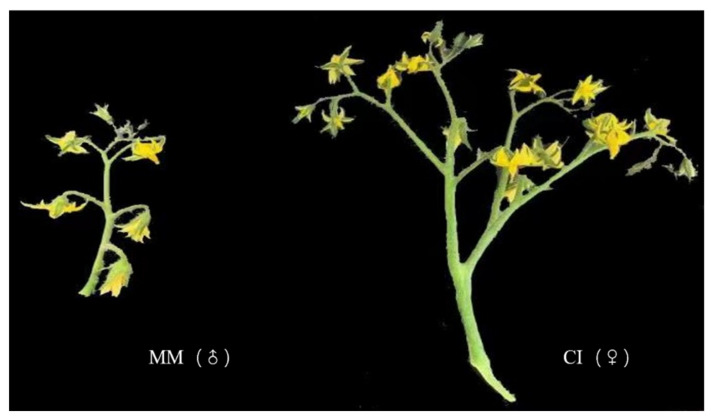
Appearance of the parental materials used for transcriptome analysis. MM (Moneymaker) was used as the male parent, and CI (Compound Inflorescence) was used as the female parent.

In this study, the two varieties were Moneymaker (identified as MM in the tran-scriptomic analysis, where MM corresponds to Control in the raw sequencing data) and the homozygous inbred line CI (represented by CI in the transcriptomic analysis, where CI corresponds to Treat in the raw sequencing data). The homozygous inbred line CI is compound inflorescence but MM is single inflorescence. Two plants showing good growth and no diseases or pests were selected from each material (both provided by the Tomato Research Group of Northeast Agricultural University) (Figure 10). In a greenhouse at the Horticulture Station of Northeast Agricultural University in Harbin, China, tomato seeds are sown in pots filled with soil and grown under controlled con-ditions (16 h of light, 25 °C, 50% relative humidity). All plants were kept at 25 °C under 50% relative humidity. Flower buds of the two different materials were collected once during each of the early, middle and late stages of inflorescence differentiation, with three replicates.

### 4.2. RNA Extraction and Transcriptome Sequencing

In this study, we sequenced the inflorescence transcriptomes of two different tomatoes using the DNA Nano Ball Sequence (DNBSEQ) platform. A total of 18 samples were analysed, and each sample yielded an average of 6.64 g of data. The reference genome was the NCBI SL3.0 assembly. Using the RNAprep Pure Plant Kit (Thermo Fisher, New York, NY, USA), total RNA was extracted from a total of 18 samples in each group for real-time quantitative PCR (qRT-PCR) and RNA sequencing (RNA-seq) analyses [67,68]. RNA-seq was performed by BGI Tech, Shenzhen, China, via the following steps: mRNA was isolated and purified from total RNA using an oligo (dT) primer. The purified mRNAs were used to construct 18 transcriptome libraries, which were then sequenced using probe-anchored polymerization techniques in DNBseq machines [69].

### 4.3. RNA-Seq Read Mapping and DEG Identification

According to the results of HISAT2 alignment, the expression of genes was calculated using the FPKM algorithm based on the read number of uniquely aligned genes and the total read number of uniquely aligned reference sequences [70]. Through the comparison of gene expression among varieties, some DEGs were screened for further enrichment analysis. The read count data representing the number of reads contained in the transcript were used as the input data in the analysis of differential gene expression. As part of the analysis, DESeq2 software [71] was used to perform the normalization of the read count data and calculate the *p*-values of significant differences and the different multiple fold-change values. DEGs were defined as genes with a log2FC change ≥ 1 and FDR value ≤ 0.05. Finally, multiple hypothesis testing was conducted for correction, and the threshold value of the *p*-value was controlled by using the false discovery rate (FDR) to avoid false positives.

### 4.4. Functional Annotation and DEG Enrichment Analysis

The functions of the DEGs were determined via the Phyper (https://en.wikipedia.org/wiki/Hypergeometric_distribution, accessed on 8 July 2021) enrichment analysis of important Gene Ontology (GO) and Kyoto Encyclopedia of Genes and Genomes (KEGG) categories. *p*-Values ≤ 0.05 were defined as the threshold, and DEGs that met this condition were defined as significantly enriched. The KEGG pathway database is the main public database focused on pathways. Sequences were aligned to the KEGG database, significant enrichment analysis was performed, the hypergeometric test was applied, and the pathways with Q values ≤ 0.05 were defined as showing significant enrichment in DEGs. GO function and KEGG pathway enrichment analyses were performed on the selected differentially expressed genes (DEGs) to obtain the main biological functions of differentially expressed proteins and the main biochemical metabolic pathways involved [72].

### 4.5. MapMan Analysis of DEGs

MapMan is software for plant-specific, mostly manually organized, pathway analysis [73]. The expression levels of DEGs in the comparison groups of MM_E vs. CI_E (MM_E vs. CI_E corresponds to Control1 vs. Treat1 in the raw sequencing data), MM_M vs. CI_M (MM_M vs. CI_M corresponds to Contro2 1 vs. Treat2 in the raw sequencing data) (https://www.plabipd.de/query_view.ep, accessed on 8 July 2021). MapMan provides a good visual interface for mapping presentation data directly onto pathway maps and generating heat maps, bar charts, and line charts for the comparison of presentation trends at different time points. MapMan further provides improved gene functional classification and comprehensive pathway maps. MapMan can be used for both model and nonmodel organisms. MapMan software was used to summarize and analyze the effective pathways affecting tomato inflorescences.

### 4.6. Weighted Gene Co-Expression Network Analysis (WGCNA)

The basic strategy of WGCNA is to mine genes with similar expression patterns and to define them based on a module algorithm [74]. Genes with similar expression patterns are likely to be closely coregulated or functionally closely related, or to be members of the same signaling pathway or process, with specific physiological significance. Compared with DEG analysis, WGCNA can provide more information, and microarray data can be more completely expressed by considering the relationships among the measured transcripts, which can be evaluated by the paired correlations between gene expression profiles [75]. WGCNA starts at the level of thousands of genes, identifies gene modules of interest, and finally uses in-module connectivity (gene importance) to identify key genes for further validation. Rather than associating thousands of genes with the characteristics of a microarray sample, the analysis focuses on the relationships between only a few (usually fewer than 10) modules and the characteristics of the sample. For this purpose, feature gene significance (the correlation between the sample feature and the feature gene) and the corresponding *p*-value for each module are calculated.

### 4.7. qRT-PCR Analysis

The reliability of RNA-seq data was detected by real-time fluorescence quantitative PCR. In this experiment, 14 DEGs were randomly selected, primers for these genes were designed according to the NCBI database, and a Reverse Transcriptase 1st Strand cDNA Synthesis Kit (TaKaRa) was used to synthesize cDNA for fluorescence quantitative qPCR analysis with the PowerUp SYBR Green Master Mix kit (ABI). Gene-specific primers designed in the NCBI database were used for real-time fluorescence quantitative PCR to analyze inflorescence samples at different developmental stages and quantify their relative expression levels. Three technical replicates were performed for each biological replicate. After the reaction, the original data, amplification curve, melting curve and other information were derived from the quantification software for analysis, and the relative expression map of the sample genes was obtained.

### 4.8. Determination of Endogenous Hormones

The effects of plant hormones on the growth of tomato inflorescences were detected. A 0.1 g sample was weighed, ground in a mortar, added to 1 mL of precooled 70–80% methanol solution (pH = 3.5), and extracted overnight at 4 °C. Then, the sample was centrifuged at 4 × 12,000× *g* for 10 min, and 0.5 mL of a 70–80% methanol solution was added to the residue. After centrifugation, the supernatant was removed; centrifugation was then performed again, and the supernatants were combined. Vacuum evaporation to 1/3 of the original volume was performed at 40 °C, and an equal volume of petroleum ether was added. The mixture was left to stand after stratification, and extraction and decolorization were repeated 2–5 times. Next, triethylamine was added, the pH was adjusted to 8.0, PVPP was added, and shock incubation was conducted for 20 min. Thereafter, centrifugation was conducted, the supernatant was collected, and the pH was adjusted to 3.0 with hydrochloric acid. Extraction with ethyl acetate was performed 3 times, after which the extract was combined with the ester phase and evaporated at 40 °C under reduced pressure until dry. The mobile phase solution was subjected to eddy oscillation and dissolved, after which it was filtered through a needle filter for testing.

## 5. Conclusions

In conclusion, to explore the factors influencing the formation of different types of inflorescences in tomato, two materials with different inflorescence types, MM and 20,965 (CI), were used for transcriptome sequencing. The analysis of large amounts of data showed that plant hormone transduction and metabolism pathways had important effects on the formation of tomato compound inflorescences. In addition, the cell cycle, photosynthesis, circadian rhythm and carbon metabolism pathways were also closely related to the formation of tomato compound inflorescences. To explore the specific factors influencing the formation of different inflorescence shapes more clearly, we screened 40 genes related to inflorescences and found that the mutation of the *S*, *AN* and *FA* genes in the late stage was the main reason for the formation of compound inflorescences. The selected genes identified herein are largely consistent with previous studies, so the reasons for the formation of compound inflorescences in our tomato material are basically the same as in cherry tomato.

## Figures and Tables

**Table 1:** Statistics of the tomato transcriptome database.

Sample	Total Raw Reads (M)	Total Clean Reads (M)	Total Clean Bases (Gb)	Clean Reads Q 20 (%)	Clean Reads Q 30 (%)	Clean Reads Ratio (%)
MM_E1	45.57	44.22	6.63	97.9	94.07	97.04
MM_E2	47.33	45.43	6.81	97.89	94.08	95.98
MM_E3	45.57	43.92	6.59	97.97	94.34	96.37
MM_M1	44.78	43.54	6.53	97.85	93.97	97.24
MM_M2	45.57	44.16	6.62	97.81	93.87	96.89
MM_M3	45.57	44.4	6.66	97.97	94.28	97.43
MM_L1	45.57	43.95	6.59	97.87	94.03	96.44
MM_L2	45.57	44.32	6.65	97.91	94.13	97.24
MM_L3	45.57	44.41	6.66	97.68	93.5	97.44
CI_E1	45.57	43.95	6.59	97.27	92.66	96.43
CI_E2	47.33	45.02	6.75	97.45	93.17	95.13
CI_E3	45.57	44.35	6.65	97.3	92.75	97.31
CI_M1	45.57	44.09	6.61	97.41	93.03	96.75
CI_M2	45.57	44.25	6.64	97.5	93.28	97.09
CI_M3	45.57	44.32	6.65	97.34	92.82	97.25
CI_L1	45.57	44.23	6.63	97.39	92.99	97.06
CI_L2	45.57	44.34	6.65	97.28	92.68	97.28
CI_L3	45.57	44.03	6.6	97.33	92.82	96.6

MM_E indicates early inflorescence development, and MM_E1, MM_E2, and MM_E3 are three samples of Moneymaker inflorescences. MM_M indicates metaphase of inflorescence development, and MM_M1, MM_M2 and MM_M3 are three samples from metaphases of Moneymaker inflorescences. MM_L indicates late inflorescence development, and MM_L1, MM_L2 and MM_L3 are three samples from late inflorescence development in Moneymaker. CI (Compound Inflorescence) represents the 20,965 homozygous inbred lines. CI_E indicates early inflorescence development; CI_E1, CI_E2, and CI_E3 are three samples from the early stage of inflorescence development in CI_E. CI_M represents metaphase of inflorescence development, and CI_M1, CI_M2, and CI_M3 are three samples from the middle stage of inflorescence development in CI_M. CI_L indicates metaphase of inflorescence development, and CI_L1, CI_L2, and CI_L3 are three samples from late inflorescences of CI_L.

**Table 2:** Affect-related genes of tomato inflorescence traits.

Pathway	Gene ID	Gene Symbol	Log2 Fold-Change	
			MM_E vs. CI_E	MM_M vs. CI_M
Metabolic pathways	101260940	*FZY4*	5.8	2.65
Metabolic pathways	101251255	*ACO2*	1.58	0.56
Metabolic pathways	108491835	*Cab-3C*	1.86	1.68
Metabolic pathways	543502	*LIN6*	1.69	1.12
Metabolic pathways	544150	*DFR*	1.35	1.14
Metabolic pathways	544149	*CHI3*	1.25	1.26
Metabolic pathways	543959	*TPX2*	1.07	1.08
Metabolic pathways	101255734	*AnthOMT*	2.13	1.61
Metabolic pathways	543964	*PSY2*	6.66	3.36
Metabolic pathways	543819	*PLDa1*	3.57	8.38
Metabolic pathways	101252616	*PMK2*	5.19	4.14
Metabolic pathways	101265056	*CMT3*	1.63	5.4
Metabolic pathways	543536	*GSH1*	5.03	4.94
Metabolic pathways	544258	*GABA-TP3*	2.66	2
Plant hormone signal transduction	101055578	*CYP735A2-TP3*	4.83	3.56
Plant hormone signal transduction	543553	*GA20ox1*	1.55	1.61
Plant hormone signal transduction	543712	*EREB*	2.86	4.84
Plant hormone signal transduction	543887	*FUL2*	0.87	1.38
Plant hormone signal transduction	101251833	*GH3-4*	1.25	2.46
Plant hormone signal transduction	543503	*GA3ox1*	2.92	1.02
Plant hormone signal transduction	543544	*IAA17*	1.89	2.85
Plant hormone signal transduction	100191111	*LOC100191111*	1.55	1.99
Plant hormone signal transduction	100736541	*SLAX5*	1.21	1.73
Plant hormone signal transduction	101055555	*IAA35*	1.65	1.04
Plant hormone signal transduction	101251833	*SIGH3.4*	1.25	2.47
Plant hormone signal transduction	101267832	*ERF68*	1.89	1.1
Plant hormone signal transduction	543644	*TKR*	0.2	1.15
Cell cycle	101250197	*cdc20-1*	0.55	1
Cell cycle	544248	*pcna*	1.23	1.06
Cell cycle	543730	*cycB1.1*	1.02	1.16
Circadian rhythm	101246029	*SFT*	0.6	0.39
	543584	*Ls*	0.6	5.85
	101253723	*TMF*	1.32	7.29
	543707	*MADSMC*	−1.22	−1.89
	543884	*MADS1*	0.44	1.24
	100301925	*AN*	2.87	4.12
	543630	*FA*	2	1.38
	543703	*BLIND*	2.81	3.57
	543770	*TM29*	−1.62	−0.18
	100240705	*S*	0.43	1.57

## Data Availability

The raw sequencing data of this article are stored in the NCBI Sequence Read Archive under accession number GSE163914.

## Supplementary Materials

The following are available online at https://www.mdpi.com/article/10.3390/ijms222212548/s1.
