# Supplementary material for: Comparative Genome Analysis of Genes Regulating Compound Inflorescences in Tomato
Source: Int J Mol Sci. 2021 Nov 21;22(22):12548. doi: 10.3390/ijms222212548 (PMC8623504; doi:10.3390/ijms222212548)
Supplement: Supplementary file 1 [file ijms-22-12548-s001.zip › ijms-1457803-supplementary.pdf]

Table S1. Gene ontology categories of DEGs.

GO_CFP Term Level2	GO_CFP Term Level1	Num
cellular process	biological_process	108
metabolic process	biological_process	93
biological regulation	biological_process	71
regulation of biological process	biological_process	71
response to stimulus	biological_process	39
developmental process	biological_process	38
cellular component organization or biogenesis	biological_process	38
reproduction	biological_process	34
reproductive process	biological_process	34
multicellular organismal process	biological_process	31
positive regulation of biological process	biological_process	27
negative regulation of biological process	biological_process	22
signaling	biological_process	18
multi-organism process	biological_process	12
cell proliferation	biological_process	10
rhythmic process	biological_process	7
growth	biological_process	3
localization	biological_process	2
immune system process	biological_process	1
cell	cellular_component	100
cell part	cellular_component	100
organelle	cellular_component	97
protein-containing complex	cellular_component	68
organelle part	cellular_component	55
membrane-enclosed lumen	cellular_component	28
supramolecular complex	cellular_component	4
cell junction	cellular_component	1
membrane	cellular_component	1
symplast	cellular_component	1
binding	molecular_function	87
catalytic activity	molecular_function	39
molecular function regulator	molecular_function	16
transcription regulator activity	molecular_function	11
molecular transducer activity	molecular_function	2

Table S2A. KEGG channel data of DEGs were analyzed in MM_E-VS-CI_E comparison.

KEGG Pathway Term ID	KEGG Pathway Term Desc	KEGG Pathway Term Level1	Term Candidate Gene Num	P value	Q value
001100	Metabolic pathways	Metabolism	108	0	0.1
000940	Phenylpropanoid biosynthesis	Metabolism	18	0	0.18
001110	Biosynthesis of secondary metabolites	Metabolism	66	0	0.18
003030	DNA replication	Genetic Information Processing	7	0	0.18
000360	Phenylalanine metabolism	Metabolism	9	0	0.23
000945	Stilbenoid, diarylheptanoid and gingerol biosynthesis	Metabolism	6	0.01	0.27
000130	Ubiquinone and other terpenoid-quinone biosynthesis	Metabolism	6	0.01	0.28
000960	Tropane, piperidine and pyridine alkaloid biosynthesis	Metabolism	5	0.01	0.28
001120	Microbial metabolism in diverse environments	Metabolism	24	0.01	0.28
003460	Fanconi anemia pathway	Genetic Information Processing	6	0.01	0.28
004111	Cell cycle - yeast	Cellular Processes	9	0.01	0.28
005212	Pancreatic cancer	Human Diseases	4	0.01	0.28
001200	Carbon metabolism	Metabolism	18	0.02	0.33
003430	Mismatch repair	Genetic Information Processing	5	0.02	0.33
004110	Cell cycle	Cellular Processes	10	0.02	0.33
004113	Meiosis - yeast	Cellular Processes	7	0.02	0.33
000941	Flavonoid biosynthesis	Metabolism	6	0.02	0.33
004710	Circadian rhythm	Organismal Systems	5	0.03	0.4
000904	Diterpenoid biosynthesis	Metabolism	4	0.04	0.5
004530	Tight junction	Cellular Processes	6	0.04	0.5

Table S2B. KEGG channel data of DEGs were analyzed in MM_M-VS-CI_M comparison.

KEGG Pathway Term ID	KEGG Pathway Term Desc	KEGG Pathway Term Level1	Term Candidate Gene Num	P value	Q value
000040	Pentose and glucuronate interconversions	Metabolism	24	0.00	0.00
001100	Metabolic pathways	Metabolism	143	0.00	0.01

004918	Thyroid hormone synthesis	Organismal Systems	5	0.00	0.08
000830	Retinol metabolism	Metabolism	5	0.00	0.11
000071	Fatty acid degradation	Metabolism	9	0.00	0.14
000100	Steroid biosynthesis	Metabolism	7	0.00	0.14
001110	Biosynthesis of secondary metabolites	Metabolism	82	0.00	0.18
004972	Pancreatic secretion	Organismal Systems	6	0.01	0.22
000626	Naphthalene degradation	Metabolism	3	0.01	0.25
000625	Chloroalkane and chloroalkene degradation	Metabolism	4	0.01	0.37
000350	Tyrosine metabolism	Metabolism	7	0.02	0.37
000520	Amino sugar and nucleotide sugar metabolism	Metabolism	13	0.02	0.39
000940	Phenylpropanoid biosynthesis	Metabolism	19	0.02	0.39
000073	Cutin, suberine and wax biosynthesis	Metabolism	5	0.03	0.46
000220	Arginine biosynthesis	Metabolism	5	0.04	0.56
000290	Valine, leucine and isoleucine biosynthesis	Metabolism	4	0.05	0.56
000909	Sesquiterpenoid and triterpenoid biosynthesis	Metabolism	4	0.05	0.56
001210	2-Oxocarboxylic acid metabolism	Metabolism	7	0.05	0.56
001220	Degradation of aromatic compounds	Metabolism	3	0.04	0.56
002024	Quorum sensing	Cellular Processes	7	0.04	0.56

Table S2C. KEGG channel data of DEGs were analyzed in MM_E-VS-CI_E comparison.

**KEGG Pathway Term ID**	**KEGG Pathway Term Desc**	**KEGG Pathway Term Level1**	**Term Candidate Gene Num**	**P value**	**Q value**
005322	Systemic lupus erythematosus	Human Diseases	33	0.00	0.00
005034	Alcoholism	Human Diseases	36	0.00	0.00
004111	Cell cycle - yeast	Cellular Processes	38	0.00	0.00
004110	Cell cycle	Cellular Processes	43	0.00	0.00
003030	DNA replication	Genetic Information Processing	20	0.00	0.00
004113	Meiosis - yeast	Cellular Processes	24	0.00	0.00
004914	Progesterone-mediated oocyte maturation	Organismal Systems	16	0.00	0.00
005203	Viral carcinogenesis	Human Diseases	32	0.00	0.00
003440	Homologous recombination	Genetic Information Processing	20	0.00	0.00
000040	Pentose and glucuronate	Metabolism	31	0.00	0.01

	interconversions				
002024	Quorum sensing	Cellular Processes	18	0.00	0.01
003430	Mismatch repair	Genetic Information Processing	12	0.00	0.04
000073	Cutin, suberine and wax biosynthesis	Metabolism	10	0.00	0.09
003460	Fanconi anemia pathway	Genetic Information Processing	13	0.00	0.12
000630	Glyoxylate and dicarboxylate metabolism	Metabolism	17	0.01	0.13
005202	Transcriptional misregulation in cancer	Human Diseases	10	0.01	0.26
004114	Oocyte meiosis	Cellular Processes	21	0.02	0.32
004218	Cellular senescence	Cellular Processes	18	0.02	0.32
000071	Fatty acid degradation	Metabolism	13	0.02	0.35
003410	Base excision repair	Genetic Information Processing	10	0.02	0.35

Table S2D. KEGG channel data of DEGs were analyzed in MM_E-VS-CI_E comparison.

KEGG Pathway Term ID	KEGG Pathway Term Desc	KEGG Pathway Term Level1	Term Candidate Gene Num	P value	Q value
005034	Alcoholism	Human Diseases	34	0.00	0.00
005322	Systemic lupus erythematosus	Human Diseases	28	0.00	0.00
003030	DNA replication	Genetic Information Processing	23	0.00	0.00
004914	Progesterone-mediated oocyte maturation	Organismal Systems	17	0.00	0.00
004111	Cell cycle - yeast	Cellular Processes	29	0.00	0.00
004110	Cell cycle	Cellular Processes	33	0.00	0.00
000500	Starch and sucrose metabolism	Metabolism	36	0.00	0.00
003430	Mismatch repair	Genetic Information Processing	15	0.00	0.00
000460	Cyanoamino acid metabolism	Metabolism	16	0.00	0.00
005203	Viral carcinogenesis	Human Diseases	30	0.00	0.00
005202	Transcriptional misregulation in cancer	Human Diseases	13	0.00	0.01
003410	Base excision repair	Genetic Information Processing	13	0.00	0.01
000195	Photosynthesis	Metabolism	15	0.00	0.01
000680	Methane metabolism	Metabolism	15	0.00	0.04
004113	Meiosis - yeast	Cellular Processes	17	0.00	0.04
005210	Colorectal cancer	Human Diseases	9	0.00	0.04

004540	Gap junction	Cellular Processes	7	0.00	0.06
003440	Homologous recombination	Genetic Information Processing	15	0.00	0.07
000906	Carotenoid biosynthesis	Metabolism	10	0.01	0.12
004933	AGE-RAGE signaling pathway in diabetic complications	Human Diseases	6	0.01	0.13

Table S3 The correlation of tomato inflorescence genes in MM_L-vs-CI_L

Pathway	Gene ID	Gene Symbol	Log2 Fold-Change
			MM_L-vs-CI_L
	100301925	AN	-6.52
	543630	FA	-1.6
	100240705	S	-0.76

Table S4 14 pairs of primers for qRT-PCR

Primer	5'to3'
AN	TGCAAGATTCAAGCCCCGAT
	GCAATGCCCAAAGCCTCAAA
FA	AAGCGAGAGACAAAGGGAGC
	ACGAGGTCCTCTGTTACCAC
S	TGACGTGCGAAGCAAGTAGT
	ACGTACGTGTGGTTGTAGGG
TMF	CGGGTTTTCCACCGTTTTCC
	TCGATGACGATGTTAGCGGG
SFT	CATGTGAGGGAGCGATGGT
	GGATTGTGCATGACTGTGGC
pcna	AAAGACACGACTCAGTCCCA
	ACCATCAAGGCTGACGATGG
IAA17	TGTCCCATGGGGGTAAGACT
	CCATAGCCCTTGGTGCTGAA
IAA35	ATCCATTTGAATTTTTAGCTCCTCT
	CTACGGTGGCAGAGCTTCTT
Cab-3C	GTATTGCTGGTGGGGCCTCTT
	AAAGCCTCTGGGTCATCAGC
LIN6	AGACGGGTAGATAGCGGGTT
	ACAACAGATCCAAATGGTGAGTC
GA20ox1	TGATGTCCACATGTATATACTCCCA
	GATAGCGCCTGCAAAAAGGG
GA3ox1	CTCGCTCTCCTGATGGTGTC
	TTCTGAATTATGAATTTGCCTCGT
PSY2	CTGGGTGCAGCCATTCAGAGA

DFR	AGTAGCAGATGCAAACGATCA<br>AAGATGACAGGCTGGGTTCG<br>TACAGAGGAGCAGAGTGGCT

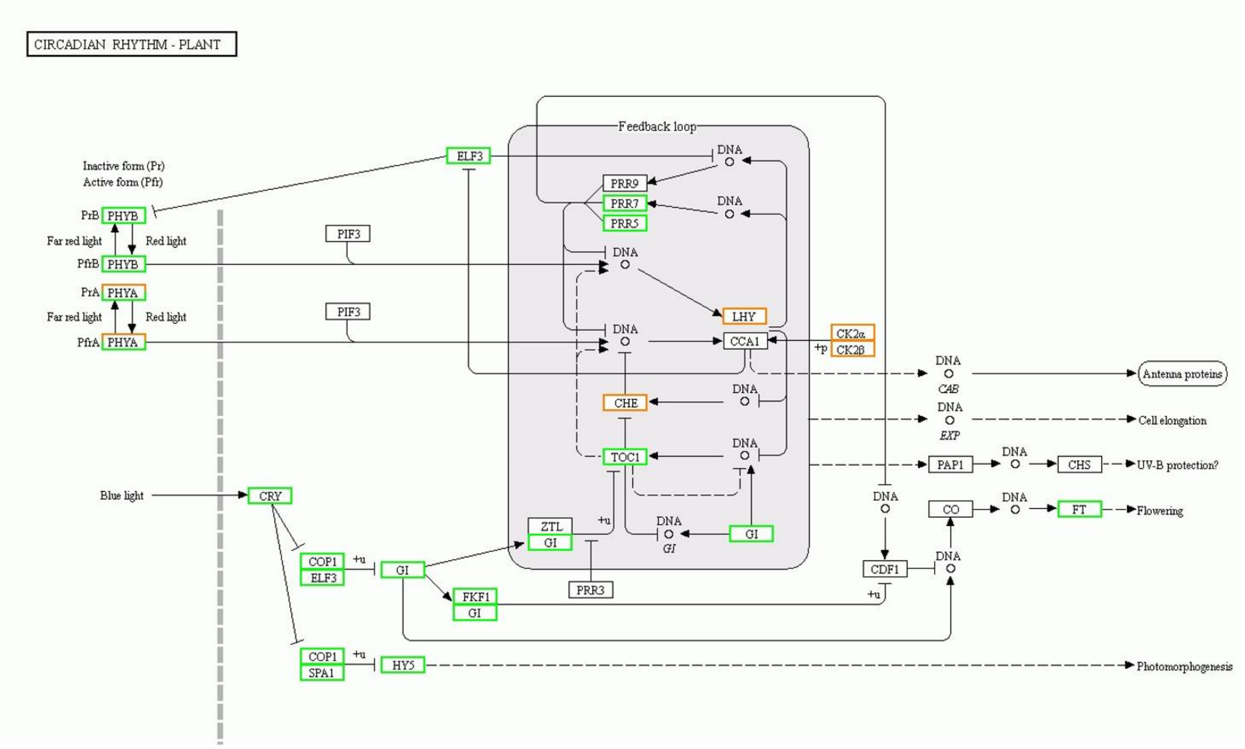

Figure S1 Introduction to the circadian rhythm pathway map
